# What is the influence of occlusal plane rotation in orthognathic surgery on upper airway volume?

**DOI:** 10.1007/s10006-026-01526-8

**Published:** 2026-02-21

**Authors:** Fábio Marzullo Zaroni, Nayara de Oliveira dos Reis, Halina Grossmann Pereira, Fernanda Aparecida Stresser, Bernardo Olsson, Delson João da Costa, José Vinicius Bolognesi Maciel, Rafaela Scariot

**Affiliations:** 1 Stomatology Department, Federal University of Paraná, 632 Prefeito Lothário Meissner Avenue Jardim Botânico, Curitiba, PR 80210-170 Brazil; 2 Federal University of Paraná, Curitiba, PR Brazil

**Keywords:** Orthognathic surgery, Occlusal plane, Upper airway, Cone-beam computed tomography

## Abstract

**Purpose:**

This prospective observational study investigates whether changes in the direction and magnitude of occlusal plane angulation influence upper airway volume in adult patients undergoing orthognathic surgery.

**Methods:**

Data were collected preoperatively and 7 days and 6 months postoperatively. Cone beam computed tomography (CBCT) was used to measure occlusal plane angulation and upper airway volume. All measurements were performed by a single, calibrated researcher trained by a gold standard specialist. Statistical significance was set at *p* < 0.05.

**Results:**

Forty-eight patients were included: 27 women (56,25%) and 21 men (43,75%), with a median age of 29 years (range 18–52). Skeletal classifications were as follows: 19 class II (39,58%), and 29 class III (60,42%). Occlusal plane rotation was counterclockwise in 24 patients (50%) and clockwise in 24 patients (52%). Rotational changes were minor (0–2°) in 24 cases (50%) and major (> 2°) in the remaining 24 cases. No significant differences were observed in rotation direction or magnitude between class II and III patients (*p* > 0.05).

**Conclusion:**

Class II patients showed a significant increase in oropharyngeal volume (*p* = 0.026), while class III patients showed an increase in nasopharyngeal volume (*p* = 0.003), regardless of occlusal plane changes. Additionally, nasopharyngeal volume increased significantly in class III patients with clockwise (*p* = 0.035) and counterclockwise (*p* = 0.037) rotations.

## Introduction

Orthognathic surgery (OS) is a range of surgical techniques used to correct dentofacial deformities, which can indirectly affect the volume of the upper airway [1]. Its principle involves surgically manipulating the bones of the facial skeleton, primarily the maxilla and mandible, to restore anatomical relationships and function [2]. This change in bone position directly impacts the upper airway. Due to its importance, maxillofacial surgeons are concerned with which bone movements and their extent most affect the dimensions of the upper airway and its repercussions [3].

In addition to anteroposterior and vertical movements of the maxillomandibular complex, rotations of the occlusal plane (OP) have become common in orthognathic surgery, either alone or in combination with other movements. Angular modifications of the OP can occur in clockwise (CW) and counterclockwise (CCW) directions [4]. CW rotation of the OP can benefit patients with a brachycephalic facial type by increasing the OP and mandibular plane angles, which increases anteroinferior facial height [5]. For patients with dolichocephalic faces, surgical correction can lead to CCW rotation of the OP, resulting in the opposite outcomes for brachycephalic patients [6]. Changing the occlusal plane is often linked to improved facial aesthetics and stability [4]. OP angle changes can modify the angulation of the mandibular plane, the exposure of the upper incisors, the anteroinferior facial height, the angulation of the upper and lower incisors, and the projection of the chin [7].

The impact of orthognathic surgery on upper airway volume varies depending on the direction and magnitude of bone movement. These dimensions may increase or decrease, impacting airflow to varying degrees [8–13]. In recent decades, cone-beam computed tomography (CBCT) and volumetric reconstruction have been essential research tools for evaluating changes in upper airway volume resulting from bone repositioning due to orthognathic surgery. The evolution of diagnostic imaging techniques, technologies, methodologies, and research has led to increasingly reliable measurement results [8, 12, 14, 15]. CBCT is particularly useful for evaluating changes before and after orthognathic surgery, providing valuable insights into the effectiveness of the surgical intervention and its impact on respiratory function [16]. CBCT processing software tools allow for the segmentation of the upper airway, providing a more comprehensive understanding of its shape and dimensions. These tools can be used in studies investigating the relationship between craniofacial morphology and upper airway dimensions. This contributes to a better understanding of how changes in jaw position after orthognathic surgery affect the upper airway [17].

Numerous studies have examined the impact of orthognathic surgery on upper airway volume and dimensions. However, this study is the first to attempt to establish a correlation between modifications in occlusal plane angulation caused by orthognathic surgery and changes in upper airway volume. For these reasons, this study aims to evaluate whether changes in the direction and magnitude of occlusal plane angulation in patients undergoing orthognathic surgery influence upper airway volume.

## Materials and methods

### Ethics

The study was approved on September 7, 2020, by the Research Ethics Committee of the Health Sciences Sector at the Federal University of Paraná according to the Declaration of Helsinki (protocol number CAAE: 38392920.2.0000.0102). Individuals invited to participate in the study received verbal information about the research. Those who agreed to participate signed an informed consent form explaining the study’s objectives, justifications, benefits, and risks.

### Sample design

This was an observational, prospective study of adult patients undergoing orthognathic surgery at the Department of Oral and Maxillofacial Surgery at the Federal University of Paraná. Participants had to be 18 years of age or older and have the autonomy to make decisions and agree to participate in the study. Data were collected at the following times: one week before surgery, seven days after surgery, and six months after surgery.

Participants were excluded from the study if they were undergoing complex craniofacial surgeries (e.g., Le Fort II and III osteotomies), had a previous history of facial surgeries or syndromes involving the development and growth of the maxilla and mandible, required orthognathic reoperation, or had a history of facial trauma.

The sample size was calculated using the open-source calculator SSPropor (http://openepi.com/SampleSize/SSPropor.htm) on the website openepi.com, version 3, and resulted in a sample size of 42 patients. The population size was considered finite (96 orthognathic surgery patients per year), with a 95% confidence level, a hypothesized 5% frequency of the outcome factor in the population [18, 19], and a design effect of 1.

### Image acquisition and measurements

CT exams were performed using an i-CAT Cone Beam 3D Imaging System (Imaging Sciences International, Inc., Hatfield, PA) with a field of view (FOV) of 16 × 13 cm, resolution of 0.25 mm, exposure time of 26.9 s, and settings of 37.07 mAs and 120 kVp. The patient was positioned with the Camper plane parallel to the ground and the sagittal plane perpendicular to it. The Camper plane extends bilaterally from the lower edge of the nasal ala to the tragus of the ear. Images were acquired at times T0 (one week before surgery), T1 (seven days after surgery), and T2 (six months after surgery).

The tomographic imaging protocol was optimized to maximize resolution, minimize radiation exposure, and adhere to the ALARA (As Low As Reasonably Achievable) principle, which avoids unnecessary radiation exposure. Participants performed the exams while seated with their mouths closed. They were instructed to relax their tongues against their front teeth, avoid swallowing, and keep their necks still. Exams were carried out with head stabilization straps to minimize the possibility of patient movement. Chin support was avoided, and special care was taken to prevent head extension, flexion, or rotation. After acquiring the images, they were processed on a workstation with i-Cat Vision software (Imaging Sciences International, Hatfield, USA), which is responsible for image reconstruction. The computed tomography data were stored in Digital Imaging and Communications in Medicine (DICOM) format and transferred to a computer station. There, they were analyzed using the free, open-source software ITK-SNAP, version 3.8.0 (http://www.itksnap.org), for measuring upper airway volume [16, 17], and 3D Slicer, version 5.0.0 (https://www.slicer.org), for measuring occlusal plane angulation [20–22].

A quality assessment was performed prior to including the CBCT images to ensure they were of adequate quality for the measurements. All CBCT scans underwent a visual assessment of head orientation and the presence of required visible anatomical structures within the field of view, as well as an evaluation of image quality. CBCT images with altered head posture (extension, flexion, or rotation) at T0, T1, or T2 were excluded. Additionally, we discarded CBCT images that were blurred due to patient movement.

All measurements and data analyses were carried out by a single investigator (FMZ) who was properly trained by an expert (JVBM) and calibrated using the gold standard. For calibration purposes, a total of 20 measurements were taken at intervals of more than seven days (10 measurements in each phase). The number of tomographic images measured (10) was approximately 15% of the estimated sample size of 70. The CBCT images analyzed were of patients undergoing orthognathic surgery (pre- and post-surgery) from a previous database. Intra-examiner reliability testing was carried out. The statistical analysis of the intra-examiner calibration demonstrated excellent reliability, with an Intraclass Correlation Coefficient (ICC) of 0.996 (0.976–0.999) for upper airway volume measurement and 0.991 (0.965–0.998) for occlusal plane angle measurement.

### Image orientation

**Fig. 1:**
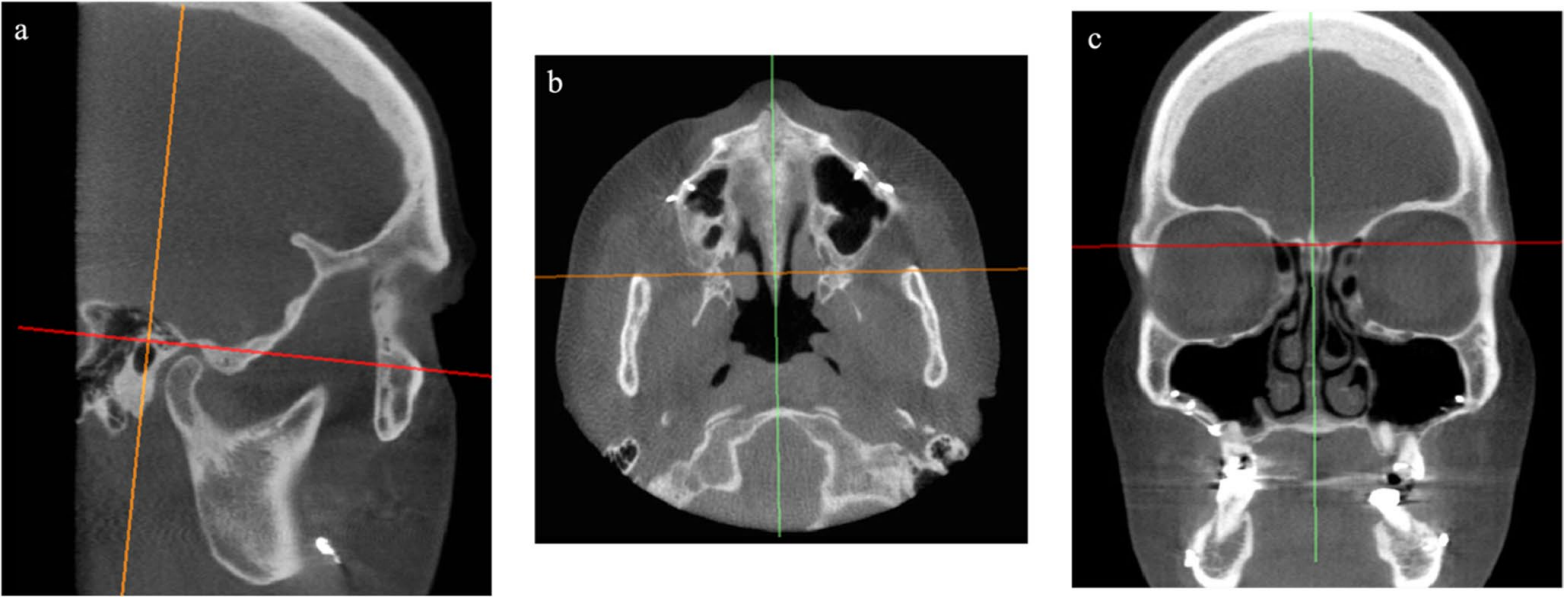
(**a**) Image orientation in sagittal view (**b**) Image orientation in axial view (**c**) Image orientation in coronal view Source: the author

After importing the DICOM files into the software, the first step was to correctly orient the image in space for standardization. (Fig. 1) The process began in the sagittal view using a line formed by the left porium and left orbitale points as a reference (Fig. 1a). Then, the image was oriented in the axial view using the anterior nasal spine, posterior nasal spine, center of the sphenoid bone, and foramen magnum as reference points (Fig. 1b). Finally, the image was oriented in the coronal view (Fig. 1c), using the frontozygomatic sutures as references [23, 24].

### Image preparation

The second step began with a semi-automatic software tool called Threshold, which classifies all pixels within a certain range (Hounsfield scale) and creates masks for different radiodensities of anatomical structures. This scale transforms the image’s different shades of gray into numerical values, allowing for greater differentiation between similar tones. The threshold sensitivity value for discriminating soft tissue from airway space was manually selected and adjusted so that the software would completely fill the airway space without under- or overfilling it. The Hounsfield scale range used was − 1024 HU to −500 HU, which is commonly used for airways, sinuses, and empty spaces. This range was established as the reference for this study.

All images were checked in coronal, sagittal, and axial views to verify that the limits of the structures of interest were correctly defined. If necessary, tools were used to fill spaces and adjust contours. Then, the anatomical region to be measured could be isolated, and a 3D volume could be created.

### Measurement of occlusal plane angle

**Fig. 2:**
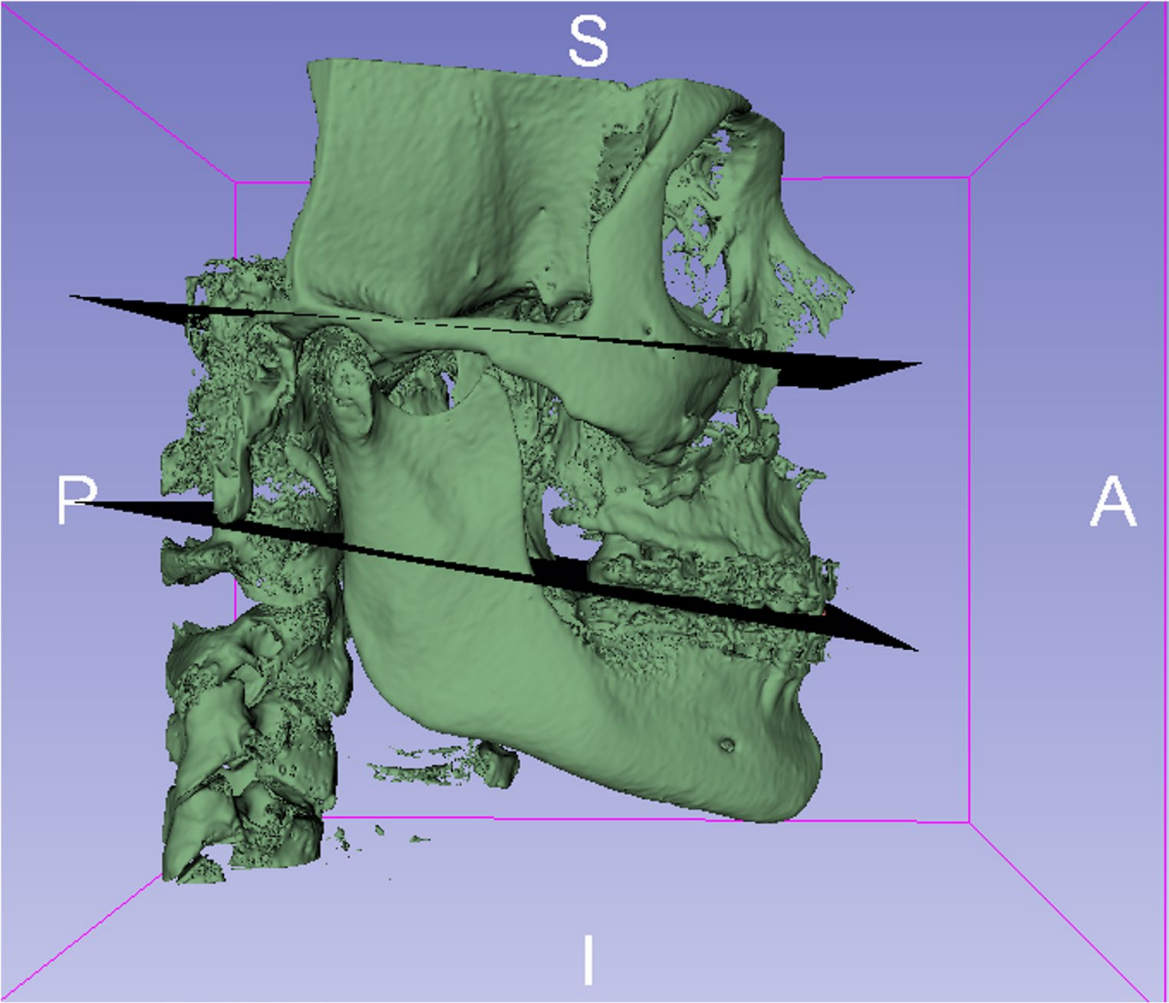
Representation of Frankfurt plane (superior) and occlusal plane (inferior). Source: the author.

The occlusal plane (OP) angle was obtained using the Frankfurt plane as a reference. The Frankfurt plane passes through the cephalometric points porion (the most superior and outer bony surface point of the external auditory meatus) and orbitale (the most inferior point on the lower edge of the orbit), as shown in Fig. 2. For this study, the right and left porion and left orbitale points were standardized to form the Frankfurt plane [25–28]. The occlusal plane (OP) was formed by connecting an anterior point located at the midpoint of contact between the upper and lower central incisors with the intercuspation points between the upper and lower right and left first molars. In cases of anterior open bite or crossbite, the functional occlusal plane described by Downs was used, which is determined by the contact points of the molars and premolars, disregarding the anterior teeth [29].

In cases of first molar absence, the second molars were used as a reference, and the second premolars were used as a last alternative. After forming the two planes, the software calculated the occlusal plane angles with the roll, pitch, and yaw components (roll: rotation on the X-axis; pitch: rotation on the Y-axis; yaw: rotation on the Z-axis). For this study, we used pitch angulation, representing the angle or inclination of the occlusal plane in the sagittal view. By comparing the occlusal plane (OP) angles between the T0 and T1 periods, we could verify the direction (clockwise or counterclockwise) and magnitude (in degrees) of the changes caused by orthognathic surgery [4–7, 30]. For statistical purposes, we considered the occlusal plane rotation direction as either clockwise (CW) or counterclockwise (CCW). Minor rotations were considered to be between 0 and 2 degrees, while major rotations were considered to be above 2 degrees, according to the median value found in the sample. Negative values ​​were considered for CCW rotations of the occlusal plane and positive values ​​for CW rotations.

### Upper airway volume measurement

**Fig. 3:**
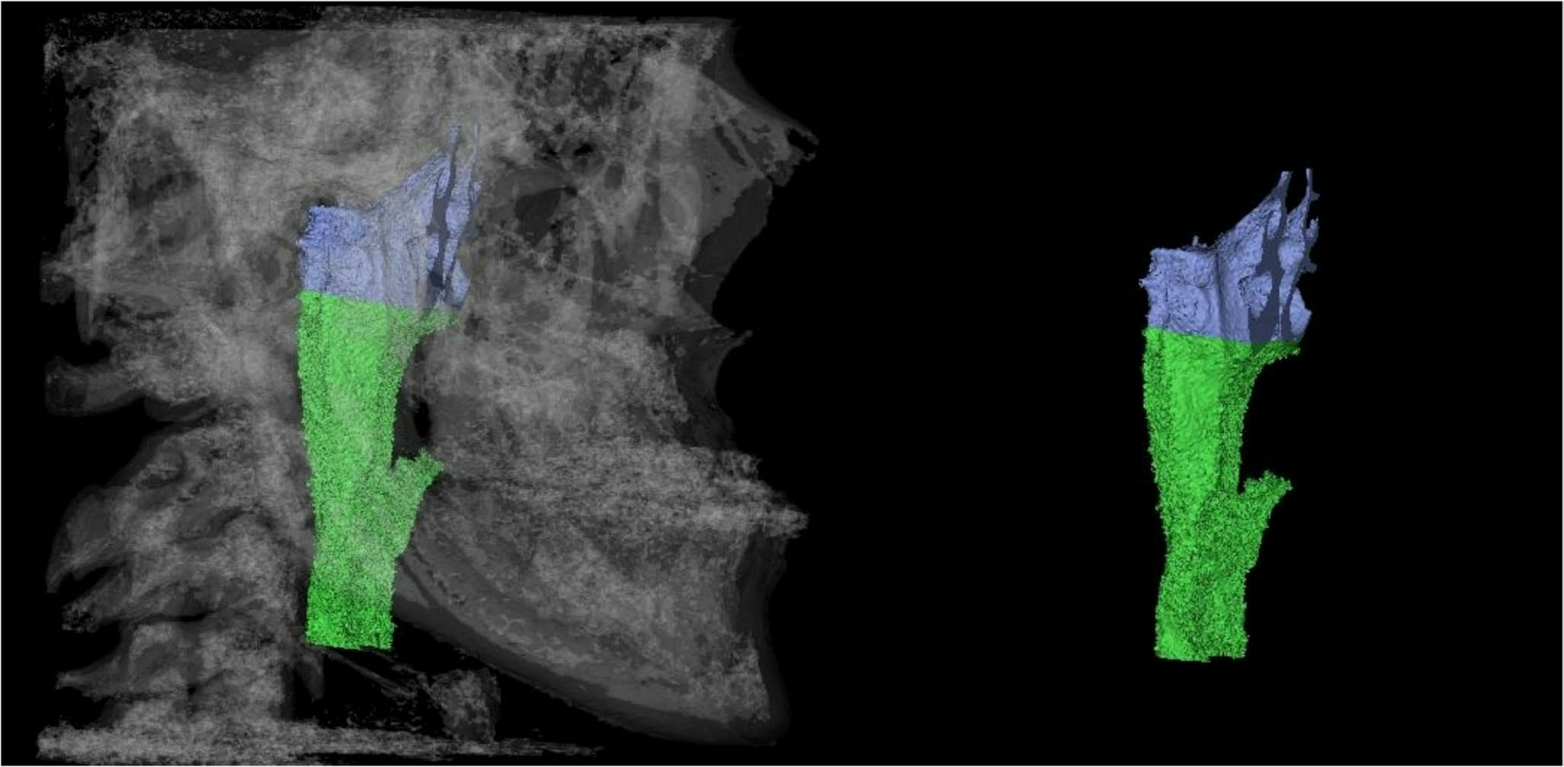
3D image of upper airway (nasopharynx and oropharynx) after segmentation. Source: the author

The upper airway image was reconstructed using 3D CBCT images of the nasopharynx and oropharynx regions. Then, a segmentation technique was used to represent the volume. This technique involves delimiting the area of interest for visualization or characterization of the anatomy through 3D reconstruction. Upper airway volume was measured using a three-dimensional model based on previously published reference points, landmarks, and planes [31, 32]. After outlining the limits of the nasopharynx and oropharynx and setting all required parameters, the investigator, the software automatically processed the upper airway volume measurements in cubic millimeters (mm³). The selected nasopharynx and oropharynx volumes were recorded. Table 1 shows the anatomical and technical limits for upper airway segmentation, and Fig. 3 exemplifies a 3D image of the upper airway volume (nasopharynx and oropharynx) after segmentation.

**Table 1:** Anatomical and technical limits of the upper airway

Region	Limits	Anatomical	Technical
Nasopharynx	Anterior	Frontal plane perpendicular to FH passing through PNS	=
	Posterior	Soft tissue contour of the pharyngeal wall	Frontal plane perpendicular to FH passing through C2sp
	Upper	Soft tissue contour of the pharyngeal wall	Transversal plane parallel to FH passing through the root of the clivus
	Lower	Plane parallel to FH passing through PNS and extended to the posterior wall of the pharynx	=
	Lateral	Soft tissue contour of the pharyngeal lateral walls	Sagittal plane perpendicular to FH passing through the lateral walls of the maxillary sinus
Oropharynx	Anterior	Frontal plane perpendicular to FH passing through PNS	=
	Posterior	Soft tissue contour of the pharyngeal wall	Frontal plane perpendicular to FH passing through C2sp
	Upper	Plane parallel to FH passing through PNS and extended to the posterior wall of the pharynx	=
	Lower	Plane parallel to FH plane passing through C3ai	=
	Lateral	Soft tissue contour of the pharyngeal lateral walls	Sagittal plane perpendicular to FH passing through the lateral walls of the maxillary sinus

*FH* Frankfort horizontal, *PNS* posterior nasal spine, *C2sp* superior–posterior extremity of the odontoid process of C2, *C3ai* most anterior–inferior point of the body of C3 Source: Guijarro-Martínez R and Swennen GR, 2013 [31]

### Statistics analysis

The independent variables in this study were the direction of rotation of the occlusal plane (clockwise [CW] or counterclockwise [CCW]) and the magnitude of movement. The dependent variable was upper airway volume.

Dentofacial deformity variables were categorized as class II or III. Follow-up periods were categorized as T0, T1, or T2. The distribution of the upper airway volume variable was assessed for normality using the Kolmogorov-Smirnov test, which showed normal distribution. Changes in upper airway volume between the preoperative and postoperative periods in class II and III patients were analyzed using a paired t-test. We compared preoperative and postoperative changes in upper airway volume in class II and III patients in different occlusal plane rotation directions using the paired t-test. An analysis of anteroposterior maxillary and mandibular repositioning in different occlusal plane rotation directions for class II and class III patients was performed using a T-test for equality of means. A significance level of 95% (*p* < 0.05) was adopted. Descriptive and inferential analyses were performed using IBM^®^ SPSS Statistics 20.0 (Statistical Package for the Social Sciences, USA).

## Results

**Fig. 4:**
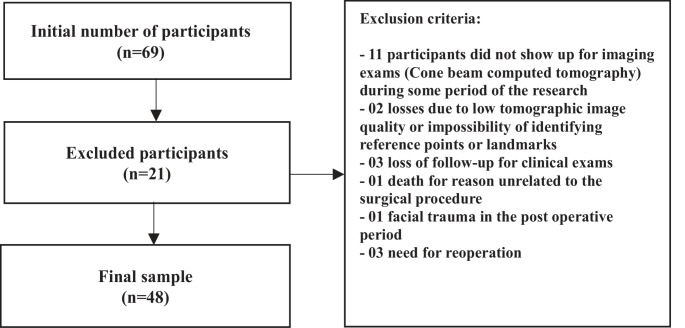
Flowchart of the sample with exclusion criteria and losses. Source: the author

Data were collected from 69 participants who agreed to participate in the research. After the end of the research period, applying the exclusion criteria and considering losses on follow-up, 48 participants remained as shown in Fig. 4. According to sex, 56.25% were women (*n* = 27) and 43.75% men (*n* = 21). The median age was 29 (18–52).

According to the type of sagittal skeletal deformity, we found 39,58% of class II (*n* = 19) and 60,42% of class III (*n* = 29). Twenty-four CCW rotations of the occlusal plane (50%) and twenty-four CW (50%) were performed. Among class II patients (*n* = 19), thirteen had CCW and six CW rotations of the occlusal plane. Class III patients (*n* = 29) had eleven counterclockwise and eighteen clockwise rotations of the occlusal plane. Rotational changes were minor (0–2°) in 24 cases (50%) and major (> 2°) in 24 cases (50%). We present the results like median (minimum – maximum) with minus sign showing CCW direction and positive values CW direction of occlusal plane rotation. Nineteen class II participants (39.6%) with − 1.26 (−10.29–2.87) and twenty-nine class III participants (60.4%) with 0.76 (−5.04–8.04).

Changes in nasopharyngeal and oropharyngeal volume between preoperative and postoperative period in class II and III patients can be seen in Table 2. There was a significant increase in the volume of the oropharynx in class II patients (*p* = 0.026) and in volume of nasopharynx in class III patient (*p* = 0.003) regardless of changes in the occlusal plane.

**Table 2:** Changes in upper airway volume between T0 (1 week before surgery) and T2 (6 months after surgery) periods for class II and class III patients (in mm3)

		Preoperative	Postoperative	Δ postop - preop	*p value
		Mean (SD)	Mean (SD)	Mean (SD)	
Class II	Nasopharynx volume	7730.64 (2712.84)	7593.86 (2477.80)	−136.78 (838.93)	0.486
	Oropharynx volume	14247.22 (4790.65)	18131.98 (7193.25)	3884.76 (6275.21)	**0.026**
Class III	Nasopharynx volume	7341.36 (3039.33)	8782.63 (4096.83)	1441.26 (2355.02**)**	**0.003**
	Oropharynx volume	20113.28 (11911.71)	20118.53 (8761.48)	5.25 (8218.28)	0.997

*Paired t-test, with significance level of 5%

SD = Standard Deviation

Bold numbers mean statistical significance

The association between preoperative and postoperative changes in upper airway volume in class II and III patients in the different directions of rotation of the occlusal plane (CW and CCW) can be seen in Table 3. It was found increased nasopharynx volume in both clockwise (*p* = 0.035) and counterclockwise (*p* = 0.037) rotations of the occlusal plane in class III patients.

**Table 3:** Comparison between T0 (1 week before surgery) and T2 (6 months after surgery) changes in upper airway volume in class II and class III patients in the different directions of rotation of the occlusal plane (in mm3)

	Class II												
	Clockwise occlusal plane rotation						Counterclockwise occlusal plane rotation						
	Preoperative Mean (SD)	Postoperative Mean (SD)		n	*p		Preoperative Mean (SD)	Postoperative Mean (SD)		n		*p	
Nasopharynx volume	8065.14 (1809.67)	7689.23 (1961.61)		6	0.774		7576.26 (3097.25)	7549.84 (2756.68)		13		0.920	
Oropharynx volume	14892.60 (4937.27)	23047.66 (5143.69)		5	0.076		13953.87 (4936.79)	16136.51 (7334.35)		11		0.307	
Total Volume	23193.67 (6347.30)	30683.72 (6738.15)		5	0.175		21848.79 (7044.78)	24032.87 (9127.45)		11		0.276	
	Class III												
	Clockwise occlusal plane rotation						Counterclockwise occlusal plane rotation						
	Preoperative Mean (SD)	Postoperative Mean (SD)	n			*p	Preoperative Mean (SD)		Postoperative Mean (SD)		n		*p
Nasopharynx volume	7968.45 (3401.74)	9324.22 (4670.71)	18			**0.035**	6315.23 (2080.12)		7896.39 (2918.39)		11		**0.037**
Oropharynx volume	21490.97 (14360.53)	20484.16 (8733.89)	18			0.648	17858.90 (6140.04)		19520.24 (9199.25)		11		0.405
Total Volume	29459.42 (17019.31)	29808.38 (12457.14)	18			0.883	24174.13 (6446.00)		27416.63 (10143.04)		11		0.149

*Paired t-test, with significance level of 5%

SD = Standard Deviation

Bold numbers mean statistical significance

The magnitude of anteroposterior maxillary and mandibular reposition (in mm) in the different occlusal plane directions of rotations for class II and class III patients is shown on Table 4. There was no significant difference between the magnitude of anteroposterior movement for the different directions of rotation of the occlusal plane between class II and III patients.

**Table 4:** Magnitude of anteroposterior movement of the jaws in relation to direction of rotations of the occlusal plane (clockwise and counterclockwise) in class II and III patients (in mm)

	Class II			Class III		
	CCW OP rotation (*n* = 13)	CW OP rotation (*n* = 6)		CCW OP rotation (*n* = 11)	CW OP rotation (*n* = 18)	
	Mean (SD)	Mean (SD)	*p	Mean (SD)	Mean (SD)	*p
Maxillary ANS_AP	0.82 (2.23)	0.66 (1.02)	0.833	4.93 (2.98)	5.98 (1.52)	0.297
Maxillary SCI_AP	1.96 (2.22)	0.67 (1.03)	0.101	5.25 (2.72)	5.26 (1.55)	0.985
Mandibular ICI_AP	6.17 (3.06)	5.93 (2.04)	0.837	0.01 (3.68)	0.71 (2.28)	0.578
Mandibular Point B_AP	7.20 (3.29)	5.58 (2.26)	0.233	0.45 (3.15)	0.13 (2.74)	0.785

*T test for equality of means, with significance level of 5%

CW = clockwise, CCW = counterclockwise, OP = occlusal plane, SD = standard deviation

Maxilla ANS_AP = anteroposterior movement registered on Anterior Nasal Spine (ANS)

Maxilla CI_AP = anteroposterior movement registered on vestibular surface of the Superior Central Incisor (SCI)

Mandible CI_AP = anteroposterior movement registered on vestibular surface of the Inferior Central Incisor (ICI)

Mandible Point B_AP = anteroposterior movement registered on mandibular Point B cephalometric landmark

## Discussion

The main findings of this study demonstrate an increase in nasopharyngeal volume in Class III participants and an increase in oropharyngeal volume in Class II participants, independent of modifications to the occlusal plane angle. Additionally, Class III participants exhibited a significant increase in nasopharyngeal volume in both clockwise and counterclockwise directions of occlusal plane rotation when comparing the T0 and T2 periods.

Changes in occlusal plane angulation, whether clockwise or counterclockwise, influence the postoperative positioning of the maxilla and mandible and may consequently affect upper airway dimensions. Understanding the relationship between occlusal plane rotation and upper airway volume is therefore essential for surgical planning. Although several studies have explored the effects of different orthognathic surgical procedures on upper airway volume, the present study is distinct in that it specifically investigates a potential direct association between occlusal plane angulation changes and upper airway volumetric responses.

Upper airway volume responds variably depending on the type, direction, and magnitude of skeletal movements performed. Variations in airway dimensions following orthognathic surgery are expected as the craniofacial complex adapts to new skeletal positions [33]. Bone repositioning can modify upper airway volume and airflow, which are directly related to breathing quality and sleep physiology. Conversely, airway narrowing or obstruction may contribute to snoring and obstructive sleep apnea (OSA) [34–36]. For this reason, identifying which surgical movements may increase or decrease upper airway volume remains a key concern for maxillofacial surgeons [3]. Consequently, numerous investigations have assessed how different orthognathic techniques, movement directions, and magnitudes influence airway volume [37–42].

Although the effects of orthognathic surgery on the upper airway are well documented, most studies have focused on overall skeletal displacement, particularly maxillomandibular advancement or setback. While it is well established that changes in maxillary and mandibular position significantly influence airway dimensions, there remains a paucity of data regarding the specific effects of occlusal plane rotation on airway volume. Furthermore, the magnitude of these rotational changes and their biomechanical relationship with airway space are not yet fully understood. This gap in the literature is clinically relevant, as occlusal plane rotation is frequently incorporated into surgical planning to improve facial aesthetics and occlusal function.

Another important aspect of this study is the assessment of upper airway volume using cone-beam computed tomography (CBCT). CBCT provides three-dimensional imaging that enables detailed visualization of airway anatomy and allows for more accurate volumetric assessment than conventional radiographs. However, concerns have been raised regarding potential inaccuracies associated with head posture, swallowing, and tongue position during image acquisition [43–45]. Despite these limitations, CBCT remains the most practical imaging modality for airway assessment when compared with magnetic resonance imaging (MRI) and multislice computed tomography (MSCT). CBCT examinations are faster and can be performed with the patient in an upright position, thereby reducing gravitational effects on the upper airway soft tissues and improving measurement accuracy [46]. Although obstructive sleep apnea events typically occur in the supine sleeping position, the objective of this study was not to evaluate OSA or sleep-related airway behavior, but rather to assess postoperative upper airway volumetric changes under standardized and reproducible imaging conditions. Accordingly, strict protocols were followed to ensure proper head positioning and stabilization during image acquisition.

The literature reports moderate to excellent reliability for CBCT-based airway measurements [47]. Although threshold selection may show variability, reliability improves with examiner training and experience, making semiautomatic segmentation more accurate than fully automated methods [48]. In this study, semiautomatic segmentation was performed with careful manual threshold selection, and airway boundaries were verified in coronal, sagittal, and axial planes. Previous studies have demonstrated the reliability and accuracy of CBCT airway measurements and consistency across different software platforms [49–51]. Examiner calibration and image quality control were rigorously conducted, resulting in excellent measurement reliability.

The impact of orthognathic surgery on upper airway dimensions has been widely reported. There is a positive relationship between the sagittal direction and extent of skeletal repositioning. Class II patients with mandibular retrognathism and reduced oropharyngeal airway dimensions are expected to benefit from mandibular advancement procedures [52]. Bimaxillary advancement has been shown to increase airway volume and improve airflow in cases of maxillomandibular deficiency [53–57]. Conversely, mandibular setback surgery may reduce airway dimensions [53, 58–60], whereas isolated Le Fort I maxillary advancement, with or without impaction, tends to increase oropharyngeal volume [61].

Studies evaluating isolated maxillary advancement, isolated mandibular advancement, and bimaxillary advancement demonstrate significant increases in oropharyngeal airway volume, with varying effects on nasopharyngeal and hypopharyngeal regions. Comparable advancements of the maxilla and mandible generally produce greater airway gains than isolated movements [13]. In Class III patients, maxillary advancement combined with mandibular setback has been associated with increased nasopharyngeal volume and reduced oropharyngeal volume [62].

Occlusal plane inclination also plays a role in airway changes. Clockwise occlusal plane rotation typically involves posterior repositioning of the mandible and chin with anterior movement of the maxilla, which may reduce airway volume by posterior displacement of the tongue and soft tissues. In contrast, counterclockwise rotation tends to advance the mandible and chin, promoting anterior displacement of the tongue and soft tissues and increasing airway volume. While some studies report minimal airway impact with counterclockwise maxillomandibular advancement combined with specific surgical techniques [4], others have demonstrated significant increases in nasopharyngeal, oropharyngeal, and hypopharyngeal volumes following counterclockwise bimaxillary advancement [41].

Previous research has suggested that normalization of the mandibular occlusal plane may predict improvement in obstructive sleep apnea following maxillomandibular advancement [63]. Other studies have highlighted the functional and aesthetic benefits of counterclockwise occlusal plane rotation in patients with OSA, although without directly measuring airway volume [34]. It is important to emphasize, however, that the objective of the present study was not to evaluate obstructive sleep apnea or related clinical outcomes, such as the apnea–hypopnea index.

In Class II participants, a significant increase in oropharyngeal volume was observed, independent of occlusal plane modification. Mandibular advancement repositions the distal mandibular segment and associated soft tissues anteriorly, including the tongue, which enlarges the oropharyngeal airway space [64–66]. The magnitude of airway increase is directly related to the extent of mandibular advancement [64], reducing airflow resistance and potentially improving breathing in patients with retrognathic mandibles.

In Class III participants, a significant increase in nasopharyngeal volume was observed regardless of occlusal plane rotation direction. Maxillary advancement, commonly performed to correct Class III deformities, advances the soft palate and increases the distance between it and the posterior pharyngeal wall, thereby enlarging the nasopharyngeal airway. Although combined maxillary advancement and mandibular setback may improve nasopharyngeal dimensions, mandibular setback can reduce oropharyngeal and hypopharyngeal volumes. The overall airway response depends on movement magnitude and individual anatomy, underscoring the need for careful preoperative planning and airway assessment.

For Class III patients, occlusal plane manipulation remains a critical surgical strategy to optimize skeletal balance, facial aesthetics, and occlusal function. Clockwise rotation with mandibular setback may reduce airway volume, whereas counterclockwise rotation with mandibular advancement tends to expand it. Despite the clinical relevance of these movements, the literature still lacks comprehensive quantitative analyses correlating occlusal plane rotation magnitude and direction with airway volumetric changes.

A limitation of this study was the inability to assess hypopharyngeal volume due to CBCT field-of-view constraints, which frequently excluded the C3 vertebral landmark [31]. Additionally, follow-up was limited by the wide geographic distribution of patients treated at this reference center, which may have contributed to participant attrition.

To date, no studies have specifically investigated the relationship between occlusal plane rotation and upper airway volume changes following orthognathic surgery. The methodology of this study was carefully designed using validated protocols to ensure reliable and reproducible results. Further research is encouraged to deepen understanding of the effects of occlusal plane manipulation on upper airway dimensions, an essential consideration in contemporary orthognathic surgical planning.

## Conclusions

There was a significant increase in oropharynx volume in Class II patients and in nasopharynx volume in Class III patients between the preoperative and postoperative periods. A significant correlation was found between the direction of rotation of the occlusal plane (OP) and changes in nasopharynx volume in Class III patients. This means that the nasopharynx increased in volume with both clockwise and counterclockwise rotations of the OP.

## Data Availability

No datasets were generated or analysed during the current study.
